# Electrical Conductivity and Dielectric Properties of Ethylene Glycol-Based Nanofluids Containing Silicon Oxide–Lignin Hybrid Particles

**DOI:** 10.3390/nano9071008

**Published:** 2019-07-12

**Authors:** Jacek Fal, Michał Wanic, Grzegorz Budzik, Mariusz Oleksy, Gaweł Żyła

**Affiliations:** 1 Department of Physics and Medical Engineering, Rzeszów University of Technology, 35-959 Rzeszów, Poland; 2 Department of Mechanical Engineering, Rzeszów University of Technology, 35-959 Rzeszów, Poland; 3 Department of Polymer Composites, Rzeszów University of Technology, 35-959 Rzeszów, Poland

**Keywords:** silica, silicon oxide, lignin, nanofluids, nanoparticles, electrical properties

## Abstract

This paper presents results of experimental investigation into dielectric properties of silicon oxide lignin (SiO_2_-L) particles dispersed with various mass fractions in ethylene glycol (EG). Measurements were conducted at a controlled temperature, which was changed from 298.15 to 333.15 K with an accuracy of 0.5 and 0.2 K for dielectric properties and direct current (DC) electrical conductivity, respectively. Dielectric properties were measured with a broadband dielectric spectroscopy device in a frequency range from 0.1 to 1 MHz, while DC conductivity was investigated using a conductivity meter MultiLine 3410 working with LR925/01 conductivity probe. Obtained results indicate that addition of even a small amount of SiO_2_-L nanoparticles to ethylene glycol cause a significant increase in permittivity and alternating current (AC) conductivity as well as DC conductivity, while relaxation time decrease. Additionally, both measurement methods of electrical conductivity are in good agreement.

## 1. Introduction

The beginning of the XXI century was a time when a constant increase in consumerism led to a continuous growth in energy demand, which resulted in raising the use of fossil fuel resources and raw minerals, and has had rough consequences for the environment. At the same time, the needs to protect the environment and the assurance of an adequate supply of energy prompts scientists to make a continuous effort to find new technological solutions in the field of energy management. A promising idea to resolve or at least reduce some problems with energy systems and their efficiency brings nanotechnology with novel material types, such as nanomaterials. One group of these materials are nanoparticles (np)—solid particles with at least one dimension lower than 100 nm. Potential applications of nanoparticles were recognised in many fields such as catalysis, biotechnology [1], electrochemical sensors, biosensors [2], environment protection [3,4], biology and medicine [5,6,7] and more [8,9,10].

Possibilities offered by materials in the nanoscale in the context of improving thermophysical properties of large scale systems were recognized by S. Choi and Eastman in 1995 [11]. They investigated thermal properties of copper nanoparticles dispersed in water and found that the addition of these nanoparticles can enhance thermal conductivity by 350%. Moreover, they used the term “nanofluid” (nf) to refer to a suspension of nanoparticles for the first time. Now, it is commonly known and recognized among researchers. Since that time, interests in nanofluids and their properties have constantly increased. Due to the huge potential of nanofluids in heat exchange systems, their thermal properties have been intensively studied. There are many papers revealing a significant increase in thermal conductivity of nanofluids containing various types of nanoparticles in different concentrations. Angayarkanni et al. [12] presented a comprehensive review paper about methods of nanofluids preparation, measurements techniques and their thermal properties. Review papers on the thermal conductivity of nanofluids have been presented by Ozerincc et al. [13] and Bashirnezhad et al. [14]. Murshed and de Castro [15] discussed in details conduction and convection heat transfer characteristics of various nanofluids in which ethylene glycol (EG) is a base fluid (bf).

Bashirnezhad et al. [16] presented an extensive review on the viscosity of nanofluids including characterization techniques, predicted models and experimental results. Sharma et al. [17] introduced an overview of impact factors such as nanoparticle type and shape, concentration, and surfactant effects on the viscosity of nanofluids. A state-of-the-art review on viscosity of nanofluids has been presented by Murshed and Estelle [18].

Other properties of nanofluids are derived from the point of view of practical applications in energy systems, especially in solar collectors. Gorjij et al. [19] conducted studies considering optical properties of nanofluids and their application in direct absorption of solar collectors. Using nanofluids in solar energy systems was also investigated by Kasaeian et al. [20] and they concluded that nanofluids can contribute to environmental and economic benefits. Possible applications of nanofluids in solar energy systems were summarized in a review paper by Mahian et al. [21].

Other benefits from using nanofluids were noted in the high voltage industry, where better efficiency of high voltage systems can be achieved thanks to improving the breakdown voltage of transformer oils as we summarized in Ref. [22].

Apart from mainstream research into the properties of nanofluids, there are other interesting and important properties of these types of fluids. Recently, more and more interest has been attached to the surface tension of nanofluids. Estellé et al. [23] introduced a wide overview on studies of surface tension of various nanofluids taking into account factors affecting surface tension and experimental correlations. They showed the correlation between surface tension and different heat transfer applications including boiling heat transfer, nucleate pool boiling, critical heat flux, heat pipes and themosyphons efficiency.

Electrical conductivity and dielectric properties of nanofluids are also aspects of mainstream of investigations. There are only several dozen papers considering electrical conductivity of nanofluids, and even less when it comes to their dielectric properties. Nanoparticles’ effect on electrical conductivity reported by researchers is usually much more visible than that in the case of thermal conductivity. Żyła et al. [24] investigated electrical conductivity of a suspension of aluminium nitride nanoparticles in ethylene glycol with a width mass fraction range of 0.05–0.20. They observed maximum enhancement in direct current (DC) electrical conductivity for the highest tested mass fraction and it was approximately 57,000%. Sarojini et al. [25] conducted electrical conductivity measurements of nanofluids with ceramic and metallic nanoparticles (CuO, Al${}_{2}$O${}_{3}$, Cu) dispersed in ethylene glycol. Their research revealed that all investigated samples showed a nearly linear increase in electrical conductivity with nanoparticles content, and the highest enhancement was observed for CuO-EG nanofluids. Moreover, they stated that Cu-EG nanofluids are in quite good agreement with the Maxwell model, when the other samples are inconsistent. One of the most significant increases in electrical conductivity in nanofluids was noted by Żyła et al. [26] for silicon nitride ethylene glycol nanofluid, with 0.1 mass fraction and it was approximately 2400 times higher than in pure ethylene glycol at 298.15 K.

The goal of this paper is the experimental investigation of the impact of nanoparticle concentration and temperature on both DC and alternating current (AC) electrical conductivity, as well as complex permittivity of ethylene glycol with dispersed silicon oxide lignin (SiO${}_{2}$-L) nanoparticles with a mass fraction from 0.01 to 0.03. The SiO${}_{2}$-L nanoparticles used in this study have been previously used to develop and manufacture complex polymers as presented elsewhere [27,28,29]. According to the best of the authors’ knowledge, there are no previous papers considering dielectric properties of nanofluids containing dispersed combined silicon oxide and lignin particles. Available papers consider nanofluids containing only silicon oxide nanoparticles and their impact on viscosity, thermal conductivity and electrical conductivity of nanofluids [30,31,32,33,34]. Results presented by Talib et al. [33] and Żyła et al. [34] indicate some possible applications of silicon oxide nanofluids for cooling in a proton exchange membrane fuel cell (PEMFC). In search of nanofluids with more favourable properties for PEMFC, silicon oxide lignin ethylene glycol nanofluids have been introduced.

## 2. Materials and Methods

### 2.1. Nanoparticles

In order to obtain a homogeneous mixture of silicon oxide (SiO${}_{2}$) and lignin (L) in a weight proportion of 1:1, appropriate amounts of both components were weighed and mixed. To reduce the size of particles and improve homogeneity, a mixture of lignin and silicon oxide was grinded for 3 h. Detailed procedure of preparation of the hybrid powder of silicon oxide and lignin and their characterisation was described elsewhere [27,29].

### 2.2. Sample Preparation

Three samples with the mass fractions of 0.01, 0.02, and 0.03, were prepared using a previously prepared mixture of silicon oxide and lignin as suspension in ethylene glycol. Firstly, the appropriate amount of powder and ethylene glycol were weighted with an analytical balance WAS 220/X (Radwag, Radom, Poland) and mechanically stirred for 30 min using a Genius 3 Vortex (IKA, Staufen, Germany). To improve homogeneity of suspension, samples were sonicated in an ultrasound water bath Emmi 60 HC (EMAG, Moerfelden-Walldorf, Germany) for 90 min and another 30 min using ultrasound probe Sonics Vibracell VCX130 (Sonics & Materials Inc., Newtown, CT, USA) at ambient temperature.

### 2.3. Measuring Methods

In order to obtain dielectric properties of silicon oxide lignin ethylene glycol nanofluids with various mass concentrations, broadband dielectric spectroscopy was applied (Concept 80, Novocontrol Technologies GmbH, Montabaur, Germany). Measurements were conducted at a controlled temperature in the range from 298.15 to 333.15 K (298.15, 313.15, 323.15, 333.15 K) and accuracy of temperature stabilization was 0.5 K. Frequency of the external electric field was changed starting from 0.1 to 1.0 MHz in 67 steps on a logarithmic scale.

**Figure 1:**
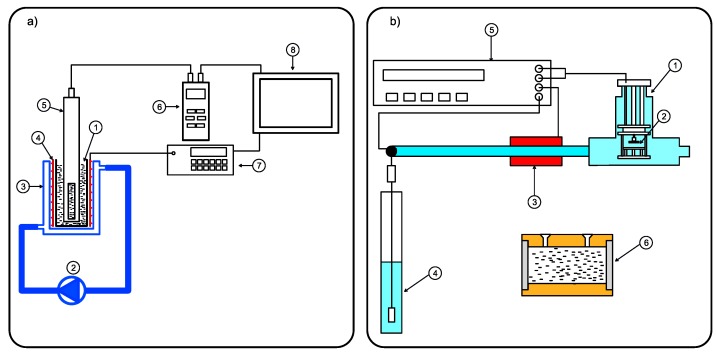
Scheme of (**a**) direct current conductivity measurement station: 1–nanofluid sample, 2–heater/refrigerator circulator, 3–water jacket, 4–heater, 5–conductivity probe, 6–conductivity meter, 7–heater controller, 8–computer with LabView software, (**b**) dielectric properties: 1–temperature stabilization chamber, 2–measuring cell holder, 3–heater, 4–column with compressed air, 5–impedance analyzer and temperature controller, 6–measuring cell.

Direct current conductivity of SiO${}_{2}$-L-EG nanofluids was measured with a conductivity meter (MultiLine 3410, WTW GmbH, Weilheim, Germany) working with a conductivity probe LR925/01 (WTW GmbH, Weilheim, Germany). To control temperature during DC conductivity measurements, a Lake Shore 335 (Lake Shore Cryotronics, Westerville, OH, USA) working with 25 Ω heater and refrigerated circulator Hanon FCH6-20 (Jinan Hanon Instruments Co., Ltd, Jinan, China) were used. A measurement station scheme is presented in Figure 1.

## 3. Results and Discussion

**Figure 2:**
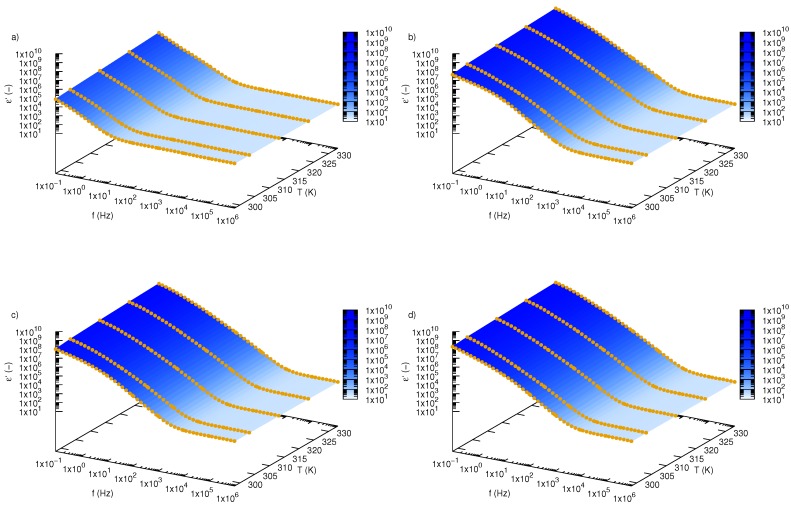
Real part of permittivity, ε’, of silicon oxide lignin ethylene glycol nanofluids as function of frequency and temperature for (**a**) pure ethylene glycol, (**b**) 0.01 mass fraction, (**c**) 0.02 mass fraction, (**d**) 0.03 mass fraction.

**Figure 3:**
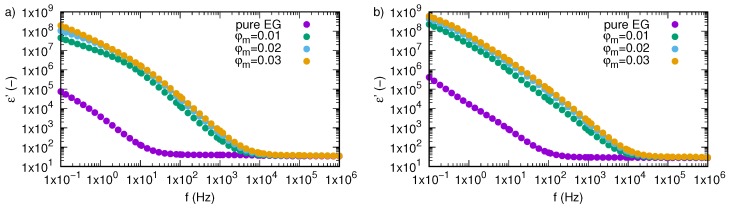
Real part of permittivity, ε’, of silicon oxide lignin ethylene glycol nanofluids as function of frequency for temperature (**a**) 298.15 K, (**b**) 333.15 K.

Figure 2 shows dependence of the real part of permittivity, ε’, of SiO${}_{2}$-L-EG nanofluids on frequency and temperature for all tested mass fractions and pure ethylene glycol. Behaviour of SiO${}_{2}$-L-EG nanofluids under alternating external electric fields can be divided into two parts. In the low frequency region, a real part of permittivity strongly depends on the frequency of the electric field, while in the second region (higher frequencies), it is almost unaffected by frequency changing. Borders between these two areas are dependent on temperature and mass concentration of nanoparticles in ethylene glycol. Comparing permittivity of pure EG (Figure 2a) with SiO${}_{2}$-L-EG nanofluids with various mass fractions (0.01—Figure 2b, 0.02—Figure 2c, 0.03—Figure 2d), it is clearly visible that the addition of SiO${}_{2}$-L nanoparticles causes an increase in permittivity and shift border of these two regions towards higher frequency. The temperature effect is clearly noticeable for each tested sample. For better presentation of impact of both temperature and nanoparticle concentration on ethylene glycol properties, experimental results for all tested concentrations for two extrema investigated temperatures (298.15 and 333.15 K) are depicted in Figure 3. Based on the chosen experimental data (Figure 3), it is obvious that the addition of nanoparticles above 1 wt.% strongly affected the increase in permittivity of ethylene glycol. Furthermore, there is a more visible effect of conductivity in low frequencies, especially at lower temperatures.

**Figure 4:**
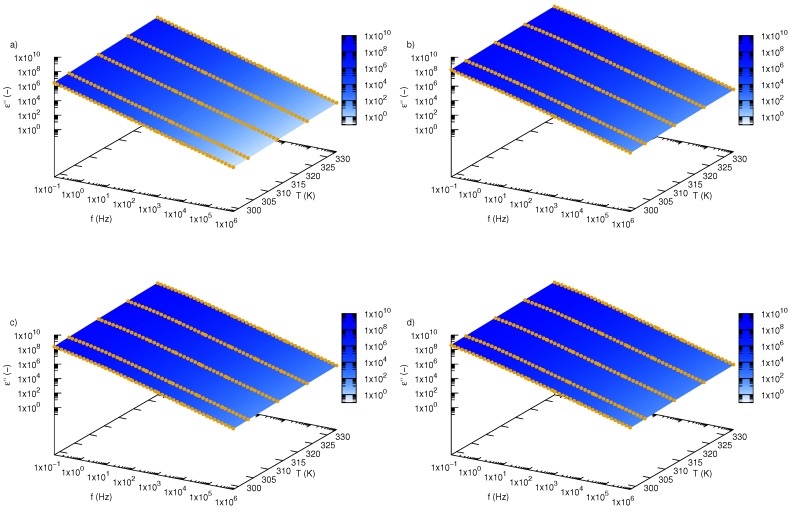
Imaginary part of permittivity, ε”, of silicon oxide lignin ethylene glycol nanofluids as function of frequency and temperature for (**a**) pure ethylene glycol, (**b**) 0.01 mass fraction, (**c**) 0.02 mass fraction, (**d**) 0.03 mass fraction.

Variation of an imaginary part of complex permittivity, ε”, as a function of frequency and temperature for SiO${}_{2}$-L-EG nanofluids and pure ethylene glycol was presented in Figure 4. The obtained experimental data create straight lines with constant slopes in all the investigated frequency range. The imaginary part of the dielectric constant is directly related with energy losses.

**Figure 5:**
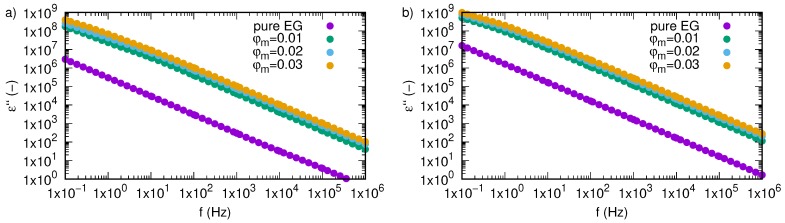
Imaginary part of permittivity, ε”, of silicon oxide lignin ethylene glycol nanofluids as function of frequency for temperature (**a**) 298.15 K, (**b**) 333.15 K.

High energy losses at low frequency are caused by free charge motion and indicate increased mobility of charge carriers. An increase in frequency causes a decrease in space charge polarization, which affects the decreasing energy losses [35]. Variation of energy losses as a function of frequency depicted in Figure 5 reflects the scale of SiO${}_{2}$-L nanoparticle effect on a real part of permittivity. Note that even small amounts of nanoparticles (1 wt.%) significantly increase values of energy losses in the whole investigated frequency range. Further increase in nanoparticles load of ethylene glycol also causes growth in the imaginary part of permittivity, but differences between successive concentrations are much smaller than that of pure ethylene glycol and the first tested samples (SiO${}_{2}$-L-EG 1 wt.%). Additionally, one percent change in concentration results in a steady increase in real part of permittivity in all tested frequency ranges, with an increase in temperature which causes an increase in energy losses.

**Figure 6:**
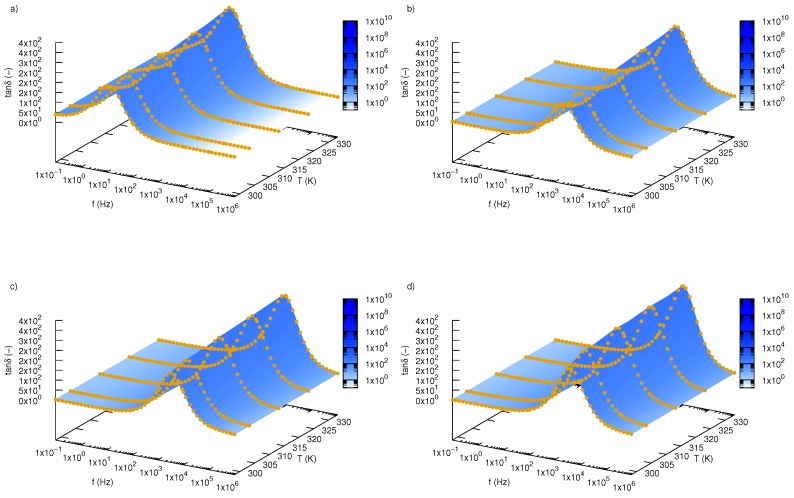
Dielectric loss tangent, tanδ, of silicon oxide lignin ethylene glycol nanofluids as function of frequency and temperature for (**a**) pure ethylene glycol, (**b**) 0.01 mass fraction, (**c**) 0.02 mass fraction, (**d**) 0.03 mass fraction.

**Figure 7:**
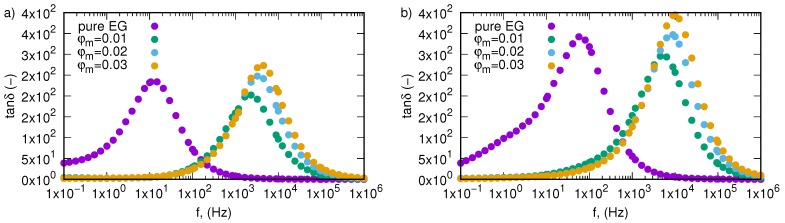
Dielectric loss tangent, tanδ, of silicon oxide lignin ethylene glycol nanofluids as function of frequency for temperature (**a**) 298.15 K, (**b**) 333.15 K.

**Figure 8:**
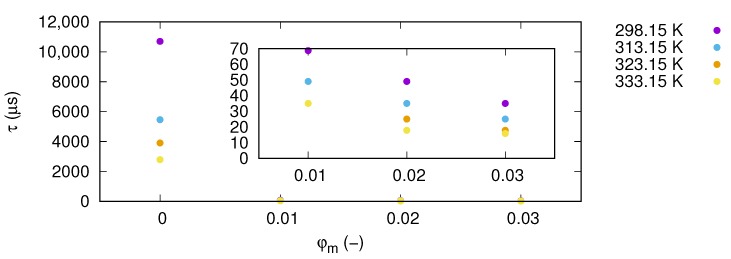
Variation of relaxation time SiO${}_{2}$-L-EG nanofluids as function of mass fraction for various temperatures. Inset presents magnification of results in mass fraction range from 0.01 to 0.03.

Dielectric loss tangents, tanδ, versus frequency and temperature are presented in Figure 6 for pure ethylene glycol and three tested fractions (0.01, 0.02, 0.03). All samples show peaks related to relaxation phenomenon. Localization of these peaks depend on the concentration of SiO${}_{2}$-L nanoparticles and temperature. As the temperature rises, one can observe a shift relaxation peak towards higher frequencies. Huge displacement of relaxation peaks has a background in increasing mass fraction of silicon oxide lignin particles in ethylene glycol, which can be seen in Figure 7 where variation of the loss tangent as a function of frequency is depicted for two temperatures 298.15 K (Figure 7a) and 333.15 K (Figure 7b). Experimental data revealed that the relaxation process for pure ethylene glycol at 298.15 K occurs at approximately 5.4 Hz and increases in temperature up to 333.15 K to cause a shift to approximately 40.8 Hz. On the other hand, addition of SiO${}_{2}$-L nanoparticles to ethylene glycol has a much stronger effect on displacement of relaxation peaks towards a higher frequency. Nanoparticle addition to base fluids causes approximately a two orders of magnitude shift of relaxation peak. Knowing relaxation frequency, one can calculate relaxation time, τ, using the following equation

(1) $$\tau =\frac{1}{2\pi f_{max}},$$

where $f_{max}$ corresponds to a frequency at the maximum loss. Values of relaxation times for individual samples in all tested temperatures are presented in the Table 1 and depicted in Figure 8. Observed changes in relaxation time indicate a very strong impact of nanoparticles on the relaxation process in ethylene glycol and its intensity is stronger with more nanoparticle load. This phenomenon is most probably related to ionic conductivity relaxation effects [36]. Increases in nanoparticle fractions cause a decrease in relaxation time, which means that the relaxation process becomes faster.

**Figure 9:**
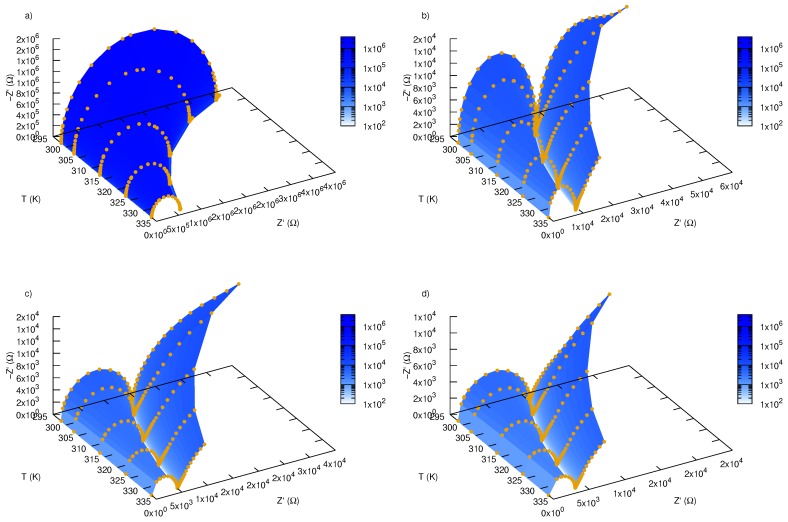
Nyquist plots of silicon oxide lignin ethylene glycol nanofluids as function of frequency and temperature for (**a**) pure ethylene glycol, (**b**) 0.01 mass fraction, (**c**) 0.02 mass fraction, (**d**) 0.03 mass fraction.

**Figure 10:**
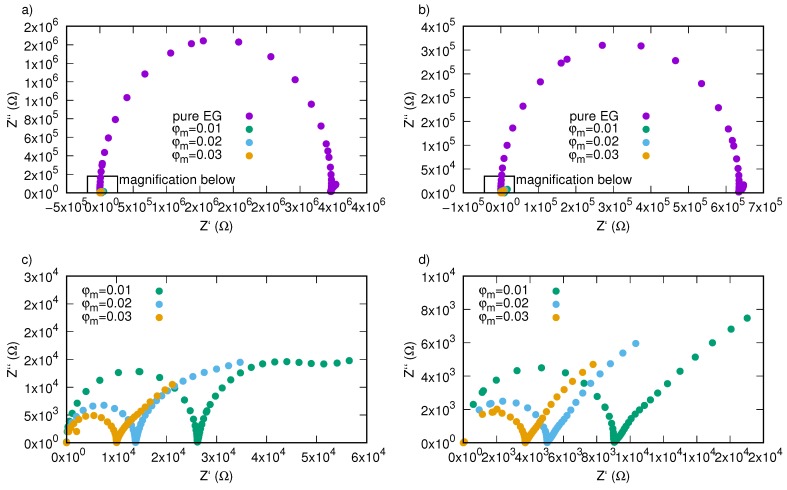
Nyquist plots of silicon oxide lignin ethylene glycol nanofluids as function of frequency for temperature (**a**) 298.15 K, (**b**) 333.15 K; (**c**) magnification of selected area from subfigure “a” for 298.15 K, (**d**) magnification of selected area from subfigure “b” for 333.15 K.

Figure 9 presents Nyquist plots for SiO${}_{2}$-L-EG nanofluids with various mass fraction as a function of frequency and temperature. One can observe that measuring data are arranged in a semicircle for all investigated samples. The radius of these semicircles is strictly related to DC conductivity and is affected by both temperature and mass concentration of nanoparticles. Temperature effects on impedance values are most visible in case of pure ethylene glycol (Figure 9a), where an increase in temperature causes significant decrease in impedance. For the other samples, effect of temperature are also visible, but the intensity is lower. For better presentation of nanoparticle impact on impedance of silicon oxide lignin ethylene glycol experimental data of both real and imaginary impedance were depicted in Figure 10, where we can see a huge decrease in the radius of semicircles caused by the addition of SiO${}_{2}$-L nanoparticles.

**Figure 11:**
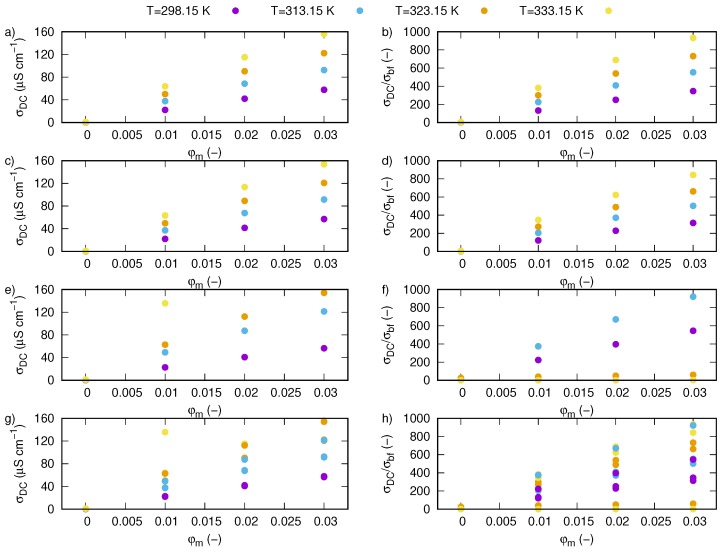
Values of DC electrical conductivity vs. mass fraction for various temperatures for silicon oxide lignin nanofluids (**a**) calculated based on Nyquist plots (Figure 9 and Equation (2)), and (**b**) their enhancement, (**c**) calculated based on AC conductivity (Figure 13) and (**d**) their enhancement, (**e**) directly measured by DC conductivity probe and (**f**) their enhancement, (**g**) comparison of results obtained with different methods and (**h**) their enhancement.

Data represented on the Nyquist plot allows to determine values of DC electrical conductivity using following formula

(2) $$\sigma_{ZZ}=\frac{t}{R_{DC}\cdot S},$$

where *t* is thickness of sample, $R_{DC}$ is direct current resistivity, designated as point of intersection impedance with axis Z’, *S* is area of sample. Values of DC conductivity calculated from Equation (2) were summarized in Table 2 and plotted in Figure 11a.

**Figure 12:**
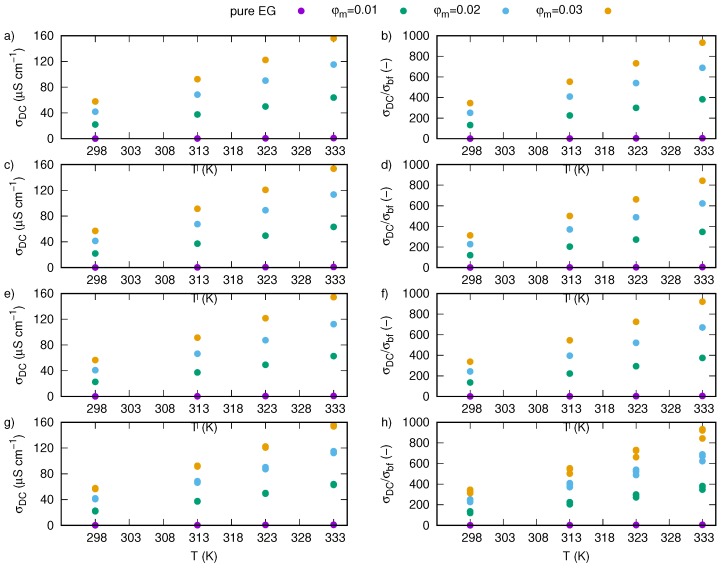
Values of DC electrical conductivity vs. temperature for silicon oxide lignin nanofluids with various mass fractions (**a**) calculated based on Nyquist plots (Figure 9 and Equation (2)), and (**b**) their enhancement, (**c**) calculated based on AC conductivity (Figure 13) and (**d**) their enhancement, (**e**) directly measured by DC conductivity probe and (**f**) their enhancement, (**g**) comparison of results obtained with different methods and (**h**) their enhancement.

Figure 11b shows enhancement of electrical conductivity as ration of electrical conductivity of nanofluid to electrical conductivity of a base fluid at 298.15 K. Calculations show that increase in mass fraction and temperature cause increase in electrical conductivity, wherein temperature effect is much smaller than mass fraction, which is visible in Figure 12a,b.

**Figure 13:**
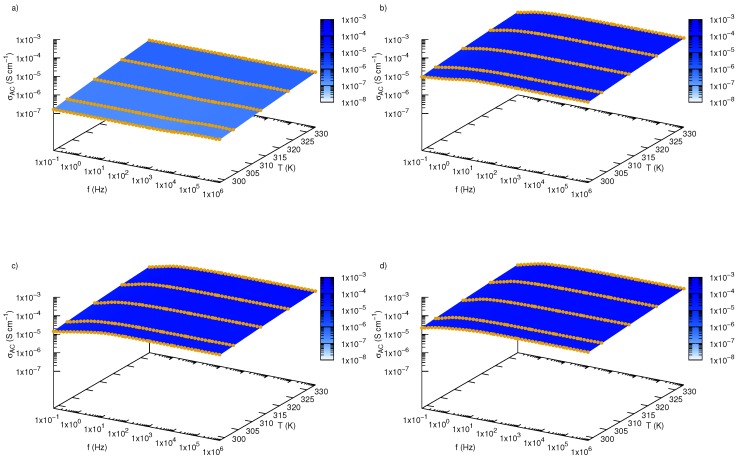
Electrical conductivity of silicon oxide lignin ethylene glycol nanofluids as function of frequency and temperature for (**a**) pure ethylene glycol, (**b**) 0.01 mass fraction, (**c**) 0.02 mass fraction, (**d**) 0.03 mass fraction.

**Figure 14:**
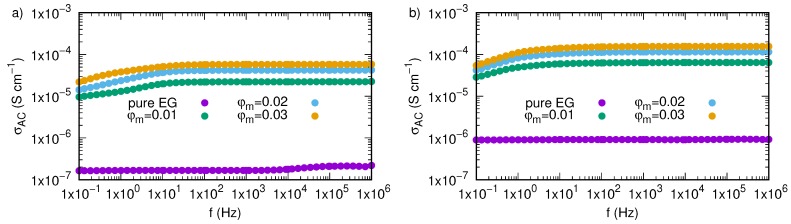
Electrical conductivity of permittivity of silicon oxide lignin ethylene glycol nanofluids as function of frequency for temperature (**a**) 298.15 K, (**b**) 333.15 K.

Dependence of AC electrical conductivity on frequency and temperature for pure ethylene glycol and nanofluids containing various mass fractions of SiO${}_{2}$-L nanoparticles were plotted in Figure 13. We have shown that temperature has an impact on all samples, and an increase in temperature affects values of AC conductivity in all investigated frequency range. In the low frequency region, there is a slightly slope caused by electrode polarisation effect. Regions with constant values of AC conductivity, not dependent on frequency, are strictly related to DC conductivity. Occurrence of regions unaffected by frequency was also presented in Figure 14, where an effect of nanoparticles is more visible. Based on these areas, values of DC conductivity were calculated as average from values of AC conductivity between 10 Hz and 1 MHz. Results of these calculations are summarized in Table 3 and plotted in Figure 11b,d, where electrical conductivity and enhancement were presented, respectively. Obtained results also revealed electrical conductivity dependence on both mass fraction and temperature, moreover, they are consistent with the obtained methods (Table 2).

Experimental results of direct current electrical conductivity of silicon oxide lignin ethylene glycol nanofluids for various mass fractions and temperatures obtained by direct measurements with conductivity probe were summarized in Table 4 and plotted in Figure 11e,f.

Experimental results show strong dependence of electrical conductivity on SiO${}_{2}$-L nanoparticles mass fraction in ethylene glycol at all tested temperatures. Maximum enhancement of electrical conductivity was noted for the highest investigated mass fraction (0.03) at 333.15 K and it was over 92,000%. The observed increase in values of electrical conductivity and their enhancement show close to linear behaviour with increasing SiO${}_{2}$-L nanoparticle mass fraction. Also, a temperature effect presents linear enhancement in electrical conductivity.

**Figure 15:**
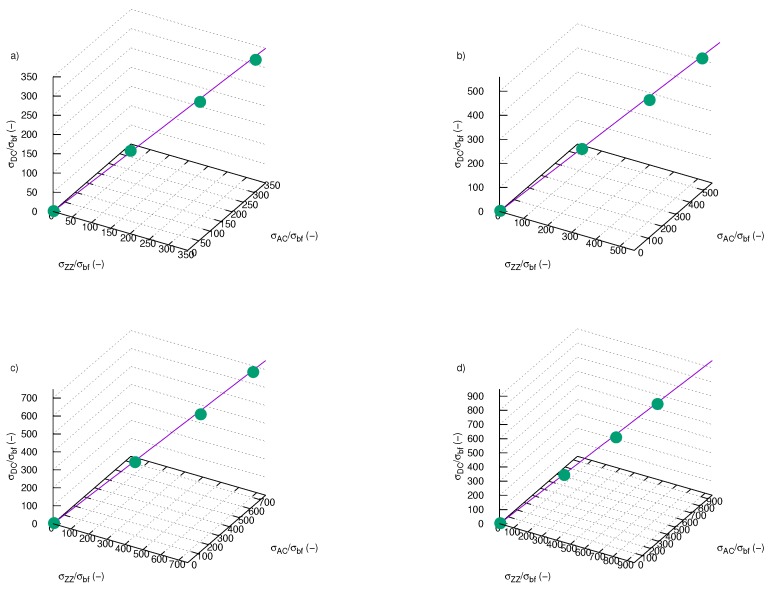
Comparison results of electrical conductivity enhancement obtained with three different methods for temperature: (**a**) 298.15 K, (**b**) 313.15 K, (**c**) 323.15 K, (**d**) 333.15 K; $\sigma_{DC}/\sigma_{bf}$—electrical conductivity enhancement from direct measurements, $\sigma_{AC}/\sigma_{bf}$—electrical conductivity enhancement calculated from plateau (Figure 13); $\sigma_{ZZ}/\sigma_{bf}$—electrical conductivity enhancement calculated from Equation (2).

Values of electrical conductivity obtained with three different methods are in good agreement with each other as presented in Figure 15, especially at low mass fractions. The higher SiO${}_{2}$-L nanoparticles load in ethylene glycol the biggest deviation from an ideal agreement. Figure 15 presents correlations between the results of DC electrical conductivity enhancement obtained with three methods for various temperatures. Values presented on each axes are related to different methods of designation electrical conductivity enhancement. Straight line represents ideal agreement between these methods, points are experimental results.

## 4. Conclusions

This paper has presented results of experimental investigation of permittivity, loss factor, AC and DC conductivity of silicon oxide lignin ethylene glycol nanofluids. We showed that, with increasing nanoparticle mass fraction dispersed in ethylene glycol, both permittivity and conductivity of samples increase. Addition of silicon oxide lignin particles to ethylene glycol causes an increase in free charge motion in samples, which results in high energy losses and significant enhancement in electrical conductivity, due to SiO${}_{2}$-L-EG nanofluids not being suitable for applications in proton exchange membrane fuel cells. At the same time, it was confirmed that an increase in temperature also caused growth in permittivity and conductivity but this effect was weaker than that in the case of increasing mass fraction. On the other hand, the effect of nanoparticles on relaxation time was also noted and an increase in nanoparticle load resulted in a significant decrease in relaxation time. Additionally, values of DC electrical conductivity obtained with different methods showed quite good compatibility with each other.

## Figures and Tables

**Table 1:** Values of relaxation time, τ, for various nanofluids concentration at temperature between 298.15 K and 333.15 K.

$\varphi_{m}$	τ, (μs)			
	298.15 K	313.15 K	323.15 K	333.15 K
0	10,700.2	5459.3	3899.5	2785.4
0.01	68.8	49.1	35.1	35.1
0.02	49.1	35.1	25.1	17.9
0.03	35.1	25.1	17.9	15.9

**Table 2:** Values of DC electrical conductivity, $\sigma_{ZZ}$, of silicon oxide lignin ethylene glycol nanofluids and their enhancement for various temperatures and mass fractions calculated based on Nyquist plots (Figure 9) and Equation (2).

$\varphi_{m}$	$\sigma_{\mathrm{ZZ}}$, ($\mu \;S\;{\mathrm{cm}}^{-1}$)				$\sigma_{\mathrm{ZZ}}$/$\sigma_{\mathrm{bf}}$, (–)			
	298.15 K	313.15 K	323.15 K	333.15 K	298.15 K	313.15 K	323.15 K	333.15 K
0.00	0.17	0.37	0.59	0.91	1.00	2.23	3.53	5.46
0.01	22.11	37.66	50.11	63.93	132.21	225.13	299.56	382.21
0.02	41.95	68.50	90.30	115.15	250.78	409.54	539.86	688.40
0.03	57.81	92.52	122.34	155.92	345.61	553.16	731.43	932.18

**Table 3:** Values of DC electrical conductivity, $\sigma_{AC}$, of silicon oxide lignin ethylene glycol nanofluids and their enhancement for various temperatures and mass fractions calculated based on AC conductivity plots (Figure 13).

$\varphi_{m}$	$\sigma_{\mathrm{AC}}$, ($\mu \;S\;{\mathrm{cm}}^{-1}$)				$\sigma_{\mathrm{AC}}$/$\sigma_{\mathrm{bf}}$, (–)			
	298.15 K	313.15 K	323.15 K	333.15 K	298.15 K	313.15 K	323.15 K	333.15 K
0.00	0.18	0.39	0.60	0.92	1.00	2.11	3.29	5.02
0.01	21.92	37.21	49.54	63.27	120.21	204.07	271.75	347.04
0.02	41.46	67.58	89.06	113.51	227.43	370.66	488.46	622.58
0.03	57.06	91.43	120.73	153.66	312.98	501.49	662.19	842.80

**Table 4:** Electrical conductivity, $\sigma_{DC}$, of silicon oxide lignin ethylene glycol nanofluids for various temperature and mass fraction measured directly.

$\varphi_{m}$	$\sigma_{\mathrm{DC}}$, ($\mu \;S\;{\mathrm{cm}}^{-1}$)				$\sigma_{\mathrm{DC}}$/$\sigma_{\mathrm{bf}}$, (–)			
	298.15 K	313.15 K	323.15 K	333.15 K	298.15 K	313.15 K	323.15 K	333.15 K
0.00	0.17	0.38	0.56	0.77	1.00	2.27	3.37	4.58
0.01	22.72	37.30	49.21	62.79	135.61	222.69	293.76	374.85
0.02	40.79	66.41	87.33	112.31	243.53	396.49	521.35	670.51
0.03	56.52	91.39	121.47	154.24	337.41	545.63	725.20	920.82

## Abbreviations

The following abbreviations are used in this manuscript:

bf	base fluid
nf	nanofluid
np	nanoparticles
EG	ethylene glycol
L	lignin
SiO${}_{2}$	silicon dioxide
DC	direct current
AC	alternating current
